# Did Androgen-Binding Protein Paralogs Undergo Neo- and/or Subfunctionalization as the *Abp* Gene Region Expanded in the Mouse Genome?

**DOI:** 10.1371/journal.pone.0115454

**Published:** 2014-12-22

**Authors:** Robert C. Karn, Amanda G. Chung, Christina M. Laukaitis

**Affiliations:** College of Medicine, University of Arizona, Tucson, Arizona, 85724, United States of America; Faculty of Biology, Spain

## Abstract

The *Androgen-binding protein* (*Abp*) region of the mouse genome contains 30 *Abpa* genes encoding alpha subunits and 34 *Abpbg* genes encoding betagamma subunits, their products forming dimers composed of an alpha and a betagamma subunit. We endeavored to determine how many *Abp* genes are expressed as proteins in tears and saliva, and as transcripts in the exocrine glands producing them. Using standard PCR, we amplified *Abp* transcripts from cDNA libraries of C57BL/6 mice and found fifteen *Abp* gene transcripts in the lacrimal gland and five in the submandibular gland. Proteomic analyses identified proteins corresponding to eleven of the lacrimal gland transcripts, all of them different from the three salivary ABPs reported previously. Our qPCR results showed that five of the six transcripts that lacked corresponding proteins are expressed at very low levels compared to those transcripts with proteins. We found 1) no overlap in the repertoires of expressed *Abp* paralogs in lacrimal gland/tears and salivary glands/saliva; 2) substantial sex-limited expression of lacrimal gland/tear expressed-paralogs in males but no sex-limited expression in females; and 3) that the lacrimal gland/tear expressed-paralogs are found exclusively in ancestral clades 1, 2 and 3 of the five clades described previously while the salivary glands/saliva expressed-paralogs are found only in clade 5. The number of instances of extremely low levels of transcription without corresponding protein production in paralogs specific to tears and saliva suggested the role of subfunctionalization, a derived condition wherein genes that may have been expressed highly in both glands ancestrally were down-regulated subsequent to duplication. Thus, evidence for subfunctionalization can be seen in our data and we argue that the partitioning of paralog expression between lacrimal and salivary glands that we report here occurred as the result of adaptive evolution.

## Introduction

Ohno proposed that gene duplications create new genetic material [1], [2]. The appearance of newly-sequenced genomes has confirmed his proposal and allowed the identification of numerous gene families that arose as the result of expansions from a single gene [3]. More recently, it has been recognized that genes can arise *de novo* from noncoding sequence and from transposon exaptation [4], [5]. In some cases, a combination of different duplication mechanisms can be involved in the same gene family expansion with the effect of accelerating the rate and extent of the expansion [6], [7]. A striking example of a shift from one duplication mechanism to another is provided by the *Androgen-binding protein* (*Abp*) genes, one of the largest gene clusters in the mouse genome and one that expanded in a relatively short period of time [8]. It is particularly important to study examples such as this because most gene duplications are probably deleterious and quickly eliminated [3], in contrast to *Abp* which underwent an extensive series of duplications.

ABP is a dimeric protein that appears to have been a mammalian evolutionary novelty [8], [9]; reviewed in [10]. It is composed of two subunits, an alpha, encoded by an *Abpa* gene, disulfide-bridged to a betagamma, encoded by an *Abpbg* gene [11]; nomenclature revised in [8]. Salivary ABP has been credited with roles in sexual selection [12]–[14] and incipient reinforcement on the house mouse hybrid zone [15]; reviewed in [10], [16]. The 3 Mb *Abp* gene region on the proximal end of chromosome 7 consists of 64 paralogs, 30 of which are *Abpa* genes and 34 *Abpbg* genes. These resulted from a dramatic expansion that began in the genome of the ancestor of the genus *Mus* around seven million years ago [8]. Most of these exist in <*Abpa*-*Abpbg*> pairs (arrows point in the 3′ direction). We have called these ‘modules’ because they appear to be the original unit of duplication [8]. We suggested that this expansion began with simple duplications but then switched to non-allelic homologous recombination (NAHR), whereby larger or smaller blocks of multiple genes were duplicated [6], [16], [17]; reviewed in [18]. The result was to accelerate the rate of expansion of the *Abp* gene family.

Because the mouse is the most widely used experimental model for studies of human physiology and disease, it is important to understand ways in which the two species differ. Like most mammals, the human genome contains only single *Abpa* and *Abpbg* genes, in stark contrast to the mouse genome with its 30 *Abpa* and 34 *Abpbg* genes expanded from five ancestral clades [8]. Moreover, both genes in the pair have been silenced in humans and the other Great Apes and so human tears are devoid of ABP. In contrast, mouse tears and saliva show potentially major ABP expression. In the case of *Abp* genes, we wish to know what role their proteins play in mouse tears. Is that role still important in human tears even though they lack ABP? If it is, what mechanism plays that role instead of ABP? It is also important to understand expression patterns of paralogs that resulted from rapid gene duplication because mechanisms such as nonfunctionalization, neofunctionalization and subfunctionalization (reviewed in [3], [5], [19]–[21]) may provide clues as to how this expansion occurred.

Before any of the questions posed above can be answered, it is important to have an accurate assessment of which of the 64 *Abp* paralogs are expressed in the lacrimal gland and secreted into mouse tears. The most comprehensive survey showed that *Abp* genes are expressed essentially only in the glands and other tissues of the head and neck of C3H mice (lacrimal glands, parotid glands, sublingual glands, submandibular glands, major olfactory epithelium, vomeronasal organ, olfactory lobes, and Harderian glands) [22]. Other regions including the brain, skin, and major organs showed no *Abp* expression, except for a small amount of *a27* and *bg27* (then called *a11* and *bg11*) in prostate and ovary. Earlier studies found no ABP in urine [23] and no behavioral preference for urine targets from *a27* congenic strains [13]. Other groups focused on lacrimal gland transcripts as a proxy for gene expression [24]. All these early studies of *Abp* expression were based on an incomplete *Abp* gene repertoire because the complete mouse *Abp* gene region would not be reported until three years later [8].

The major secretions of the face and neck are saliva and tears. Genome mouse and genome rat saliva proteomes have been reported [25], [26]. Combined with earlier transcript analyses [22] these clearly show that *a27*, *bg26* and *bg27* are the only *Abp* genes that are translated into protein in saliva [27]. It is notable that these three paralogs occur in one of the oldest clades of the five ancestral clades of *Abp* genes, the one that occupies the distal flank of the *Abp* gene region on mouse chromosome 7. By contrast, the comprehensive transcript survey in the C3H mouse found more and different *Abp* paralogs in lacrimal than salivary gland cDNA libraries, also finding transcripts of two of the three saliva subunits, *a27* and *bg27,* in lacrimal glands [22]. Lacrimal transcript data from the work of others [24], [28] can be found on NEIbank, which currently recognizes three *Abp* paralogs expressed in lacrimal glands based on cloned transcript analysis, and three paralogs found in the whole eye. These estimates are substantially lower than the estimates from the transcript study of Laukaitis et al [22].

In this study, we endeavored to resolve the question of which *Abp* genes are actually expressed in lacrimal and salivary glands and translated into proteins secreted into tears and saliva. Our reference was the entire set of 64 paralogs from the completed *Abp* gene region [8]. We used the predicted paralog coding nucleotide sequences to identify lacrimal transcripts and the predicted amino acid sequences to identify peptides from the proteome analysis. Because we encountered instances of transcripts in lacrimal gland for which we found no corresponding proteins in tears, we also performed quantitative PCR (qPCR) to determine how the transcript levels of those transcripts compared with levels of transcripts with corresponding secreted proteins. We asked how much overlap there is in expression of various paralogs in the lacrimal gland and tears with the submandibular gland and saliva. Our analyses included cDNA libraries and secretion samples from both sexes of the genome mouse (C57BL/6) so that we could identify both shared and sex-limited *Abp* gene expressions.

We discuss the significance of finding very different repertoires of *Abp* genes expressed in tears and saliva, as well as sex-limited expression of nearly one-third of the paralogs in males. We are also interested in whether it is possible to understand potential neo- and/or subfunctionalization events in such a complex gene region expansion. Could the footprints of such events survive the two-phase duplication process we have described for this dramatic gene family expansion? We propose that there may be as-yet-unidentified functions for these proteins in addition to the role proposed for salivary ABP in assortative mating and incipient reinforcement on the mouse hybrid zone in Europe [10].

## Methods

All animal manipulation was performed in accordance with University of Arizona IACUC procedures under protocol 08-138. Male and female mice of strain C57BL/6 were obtained from Jackson Laboratory (Bar Harbor, Maine). The animals used to produce cDNA libraries for transcript analysis and proteins for proteomic analysis were the C57BL/6 strain of mouse (the genome mouse) and are thus essentially clones. Therefore the animals used for the two analyses should have been essentially identical to each other and have the sequences predicted from the mouse genome for the 64 different *Abp* paralogs. Approximately 50 mice of each sex were used over a period of two years.

### Transcript analyses

RNA isolation, cDNA library construction, PCR amplification of *Abp* cDNAs and sequencing was performed as previously reported [22]. Primer sequences are shown in **S1 File in** **S1 Appendix**. Paralogs were Sanger sequenced to identify internal SNPs and check them against reference sequences from the mouse genome. Sequence alignment and phylogeny construction was performed with DNAsis Max (Hitachi).

### Tear fluid sources and treatment

Animals were anesthetized as previously described [29] and lacrimation stimulated with intraperitoneal injection of 15 ng of pilocarpine in normal saline (Sigma; [30]). Tear fluid was collected by pipetting a ten µl drop of sterile normal saline at the edge of the eyelid, waiting 5 sec and removing the fluid with the micropipetter. The process was repeated four more times. The fluid was dialyzed against ammonium bicarbonate buffer, pH 8.3. The volume was reduced ten-fold in a Speed-Vac and dialyzed a second time against the same buffer. The C57BL/6 saliva proteome has been reported [25], [26].

### Proteomics analysis

Tear fluids were provided to the Proteomics Core Facility of the University of Arizona where personnel determined the protein quantity in the samples, performed two-dimensional (2D) gel separation of the proteins (isoelectric focusing x SDS gel electrophoresis) and excised six major banding pattern regions. The proteins in each 2D gel piece were digested with trypsin. LC-MS/MS analysis of the digested proteins in the gel pieces [31] was carried out using a LTQ Orbitrap Velos mass spectrometer. Peptides were eluted onto an analytical column and separated using gradients composed of acetonitrile and formic acid. Data-dependent scanning was performed by the Xcalibur v 2.1.0 software [32] using a survey mass scan at 60,000 resolution in the Orbitrap analyzer scanning *m/*z 400–1600, followed by collision-induced dissociation tandem mass spectrometry (MS/MS) of the most intense ions in the linear ion trap analyzer. Precursor ions were selected as described in **S2 File in** **S1 Appendix**. Dynamic exclusion was used to minimize masking of low-abundance peptides by the more abundant ones. MS/MS spectra were searched against Uniprot *Mus musculus* downloaded January 11, 2012 (http://www.uniprot.org/taxonomy/10090), appended with ABP sequences provided by RCK, using Thermo Proteome Discoverer 1.3. Proteins were identified at 99% confidence with XCorr score cut-offs [33] as determined by a reversed database search. The results were displayed with Scaffold v 3.6.1 (Proteome Software Inc., Portland, OR), a program that relies on various search engine results (Sequest, X!Tandem, MASCOT) and uses Bayesian statistics to reliably identify more spectra [34], [35]. Scaffold was used to validate MS/MS-based peptide and protein identifications with the criteria described in **S2 File in** **S1 Appendix**. Protein and peptide reports are available from the authors upon request.

### Quantitative PCR analyses

Quantitative PCR was performed on an ABI PRISM 7000 using a Thermo Scientific Maxima SYBR green kit according to the instructions of the manufacturer. Primers were the same as in standard PCR. PCR conditions were adjusted to optimize efficiency and r^2^ values of standard curves. Paralog expression was compared to C57BL/6 male lacrimal total RNA for all primer sets except *Abpa27, bg27* and *bg26,* which were indexed against male submandibular RNA using relative quantitation. Standard curves were created with total RNA across the range of 0.01 ng to 10 ng.

### Data analysis

CODEML in the PAML package [36], [37] was used to assess the effect of positive selection on amino acid sites in the encoded proteins. For each gene, three different comparisons of neutral and selection models gave similar results (M1 vs. M2, M7 vs. M8, and M8A vs. M8 [36]–[38]). Model M1 (neutral) allows two classes of codons, one with *dN/dS* (sometimes shown as ω) over the interval (0,1) and the other with a *dN/dS* value of one. Model M2 (selection) is similar to M1 except that it allows an additional class of codons with a freely estimated *dN/dS* value. Model M7 (neutral) estimates *dN/dS* with a beta-distribution over the interval (0,1), whereas model M8 (selection) adds parameters to M7 for an additional class of codons with a freely estimated *dN/dS* value. A likelihood ratio test (LRT) is usually performed by taking the negative of twice the log-likelihood difference between the nested models M7 and M8 and comparing this to the χ2 distribution with two degrees of freedom. However, we used M8A (neutral), a special case of M8 that fixes the additional codon class at a *dN/dS* value of one instead of M7 in calculating the LRTs [39]. In each of the four analyses, the same guide tree was used for both M8 and M8A. Phylogenetic trees were constructed from the alignments using the program PAUP* [40] using neighbor joining (NJ) distance parameters with Jukes-Cantor correction and random-seeding. These phylogenies were used as guide trees for CODEML analysis (**S3 File in** **S1 Appendix**). The three-dimensional structures of mouse ABPBG2 and ABPBG10 were modeled using the PHYRE2 threading program, version 2.0 (http://www.sbg.bio.ic.ac.uk/phyre2/html/page.cgi?id=index; [41]), and the resulting models were visualized using PYMOL (open-source 1.2.8; http://www.pymol.org/). Sites under positive selection were mapped onto the structural models and incorporated into figures.

### Data archiving

All gene sequences have been submitted to GenBank [GenBank: KM014043 through KM014099]. All *Abp* paralogs with GenBank accession numbers and coordinates are listed in **S4 File in** **S1 Appendix** **.** Additional details of the methods are in **S2 File in** **S1 Appendix**.

## Results and Discussion

### Transcriptome and proteome analyses

The complete *Abp* gene region in the mouse genome contains 30 *Abpa* and 34 *Abpbg* genes [8]. These derive from five ancestral clades in the progenitor of the genus *Mus*. The last two duplications in this rapidly expanded gene region occurred by NAHR and doubled the number of *Abp* paralogs in the mouse genome [17]. One consequence of this recent duplication is that most of the genes in the center of the region share>99.5% identity. Another consequence is that the members of pairs *a11/a18, bg14p/bg16p, a14p/a16p, bg15p/bg17p*, *a15/a17* and *bg31p/32p* are identical, thus there are only 58 unique paralog sequences. Nonetheless, we were able to design primer sets that amplify 49 of the 58 unique *Abp* paralog sequences (84%) from genomic DNA, and to confirm that the intended paralog was amplified by identifying diagnostic SNPs.

**Figure 1:**
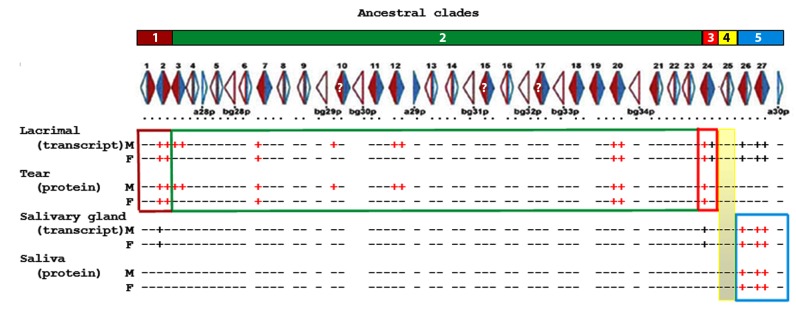
Distribution of lacrimal gland/tear and salivary gland/saliva *Abp* expressions by ancestral clade. The five ancestral clades [8] are shown at the top as numbered, colored blocks. The complete map of *Abp* genes with arrows depicting genes (*Abpa* in blue, *Abpbg* in red) appears below the clade blocks with solid filled arrows showing complete (potentially expressed) genes and open arrows showing putative pseudogenes. <*Abpa-Abpbg*> and <*Abpbg-Abpa*> modules are numbered above the linkage map [8]. Gene expressions for transcripts in lacrimal and salivary glands and proteins in tears and saliva are scored as +/− and appear below the linkage map. Those positive as both PCR products from gland cDNA libraries that could be sequenced (i.e. transcripts) and proteins in the glandular secretion appear as red + signs; those found only as transcripts that could be sequenced appear as black + signs.

We used those primers to study *Abp* expression in mouse lacrimal and submandibular glands. We detected transcripts of the following *Abp* genes in cDNA libraries of the lacrimal glands of both sexes of C57BL/6 mice: *a2, a20, a24, a27, bg2, bg7, bg20, bg24*, *bg26* and *bg27*. Transcripts of *a3*, *a12, bg3p*, *bg12* and *bg10p* (or *15p* or *17p*) were amplified from lacrimal cDNA libraries of male mice only. It was not possible to distinguish among *bg10p*, *15p* and *17p* (see below) and they will be shown hereinafter as *bg10(15/17)* without a ‘*p*’ because one or more was found as protein(s). We treat *bg3* similarly because a corresponding protein was also found. We did not find any *Abp* transcripts unique to the female lacrimal cDNA library, suggesting that there is only sex-limited expression in males and that they transcribe half-again as many paralogs in their lacrimal glands as do females. In all we found 15 different transcripts expressed in lacrimal glands (**Fig. 1** and **Table 1** **)**. Using the same primer sets in PCR experiments with submandibular gland cDNA libraries, we amplified five out of 49 paralogs: *a2* and *bg24* (both sexes, faint bands), *bg26, a27* and *bg27* (both sexes, strong bands). All of these were confirmed by identification of diagnostic SNPs by DNA sequencing. The three strongly amplified transcripts have been reported previously in salivary glands and encode subunit proteins (formerly ABPA, ABPB and ABPG; [11]) that form the A27:BG26 and A27:BG27 dimers [8].

**Table 1:** Transcriptome (lacrimal glands) and proteome (tears) identification of *Abpa* and *Abpbg* subunit expressions.

*Abp* paralog	Accession number	ABP subunit	Transcripts in sex	Transcript coverage^1^	Male protein identifications^2^	Amino acid coverage (%)^1^	Female protein identifications^2^	Amino acid coverage (%)^1^
*Abpa2*	KM014043	ABPA2	Male and female	79%	6	93%	4	87%
*Abpa3*	KM014044	ABPA3	Male only	67%	5	100%	None	N/A
*Abpa12*	KM014048	ABPA12	Male only	100%	1	44%	None	N/A
*Abpa20*	KM014051	ABPA20	Male and female	99%	6	100%	2	79%
*Abpa24*	KM014052	Transcript only	Male and female	97%	None	N/A	None	N/A
*Abpa27*	KM014054	Transcript only	Male and female	63%	None	N/A	None	N/A
*Abpbg2*	KM014055	ABPBG2	Male and female	81%	6	93%	3	93%
*Abpbg3* 3	KM014056	ABPBG3	Male only	91%	5	76%	None	N/A
*Abpbg7*	KM014057	ABPBG7	Male and female	82%	6	100%	3	72%
*Abpbg10* 3 ^,^ 4	KM014058	ABPBG10^5^	Male only	47%	1	27%	None	N/A
*Abpbg12*	KM014060	ABPBG12	Male only	93%	5	75%	None	N/A
*Abpbg20*	KM014064	ABPBG20	Male and female	81%	3	100%	1	61%
*Abpbg24*	KM014066	ABPBG24	Male and female	78%	6	88%	3	56%
*Abpbg26*	KM014067	Transcript only	Male and female	54%	None	N/A	None	N/A
*Abpbg27*	KM014054	Transcript only	Male and female	80%	None	N/A	None	N/A

1 Not including signal peptide.

2 Number of gel pieces with peptides for the protein in question.

3 Formerly designated as a pseudogene.

4 This could be *Abpbg10* or *Abpbg15* or *Abpbg17* or a combination of two or more of them.

5 This could be ABPBG10 or ABPBG15 or ABPBG17 or a combination of two or more of them.

Salivary proteomes produced for the genome mouse (C57BL/6 strain) and the genome rat (BN/SsNHsd/Mcwi strain) with multidimensional protein identification technology (MUDPIT) clearly showed that A27, BG26 and BG27 were the only *Abp* paralogs translated into protein in mouse saliva [25], [26]. For proteome analysis of lacrimal glands, we separated tear proteins on 2D gels (**S5 File in** **S1 Appendix**) and cut the gels into six pieces based on patterns of protein-staining. **Table 1** summarizes the identifications of the *Abpa*/ABPA and *Abpbg*/ABPBG transcripts and proteins, and their alignments are shown in **S6** and **S7 Files in** **S1 Appendix**. The proteins identified were: A2, A3 (male only), A12 (male only), A20, BG2, BG3 (male only), BG7, BG10 (male only), BG12 (male only), BG20 and BG24. We compared transcript and proteome data by aligning the protein sequences with the cDNAs translated into amino acid sequences (**S6 and S7 Files in** **S1 Appendix**) to identify unique amino acid residues and/or combinations of residues that would allow us to distinguish them from the putative products of the other *Abp* paralogs.

The major advantage of combining transcriptome and proteome analyses is that each provides a check on the results of the other and this often resolves problems distinguishing the expressions of closely related paralogs. Here we have described the expression of four *Abpa* and seven *Abpbg* paralogs both as transcripts in lacrimal glands (by PCR and qPCR) and proteins in tears of the genome mouse. We feel that this tripartite approach is one of the strengths of our project.

### Expression of putative pseudogenes

When the completed *Abp* gene region was analyzed and mapped, putative pseudogene status was assigned to any gene with either a non-canonical splice site, a disrupted coding region or both [8]. By this classification, the greatest number of mouse *Abp* paralogs are pseudogenes (34/64 not including *bg3* and *bg10*(*15*/*17*); **S8 File in** **S1 Appendix**). With the exception of *bg3* and *bg10*(*15/17*), we found no evidence of pseudogene expression in transcriptomes or proteomes, essentially supporting the original assignments.

Pseudogene status was originally assigned to *bg3* because it has an AG-to-AA acceptor splice site change in intron a, and to *bg10(15/17)* because it has a GT-to-GC donor splice site change in intron b. Splice site mutations in introns do not necessarily interfere with transcription and splicing [42] and some putative pseudogenes with such changes are expressed. The *bg10*(*15/17*) paralog also has a proximal 6 bp deletion, followed by a 1 bp insertion and a distal 4 bp deletion in Exon 2. It also has numerous SNPs compared to other *Abpbg* transcripts. The proximal 6 bp deletion in the coding region of Exon 2 would not change the translation reading frame, but the following 1 bp insertion does. Translation continues out of frame as far as the distal 4 bp deletion that restores the reading frame. None of these indels introduce an early termination codon, however they result in a BG10(15/17) protein shortened by three amino acid residues. These changes, along with numerous SNPs, make the amino acid sequence of BG10(15/17) quite different from the protein products of other *Abpbg* paralogs [6], [8], [17]. We identified a *bg10*(*15/17*) transcript encoding 66 bp of signal peptide (22 residues) and 123 bp (41 residues) of the N-terminus of the putative secreted protein. The protein is represented by an N-terminal peptide of sixteen residues including a diagnostic Asn28 residue as well as an 8-residue peptide from near the C-terminus of the protein, part of which is a unique Tyr-Ile-Ser-Arg (YISR) sequence.

**Figure 2:**
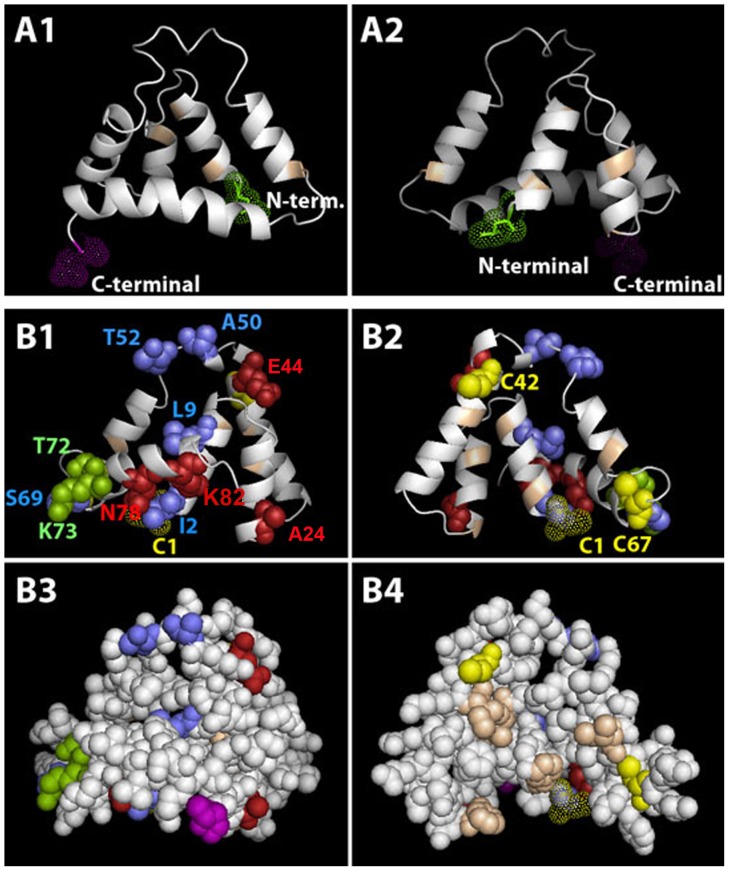
Structures of the ABPBG10 subunit (panels A1–2) and of the ABPBG2 subunit (panels B1–4) threaded on model c2ejnB with sites of interest indicated. Panels A1, A2, B1, and B2 are in cartoon format with alpha helices represented by helical ribbons and loops as thinner connecting lines. Panels B3 and B4 are space-filling models with amino acid residues as filled spheres. The conserved residues involved in ligand binding and subunit coordination in the dimer [43] are colored tan and ½-Cys residues are colored yellow. Panel A1 shows the exterior face of the ABPBG10 subunit and panel A2 shows its interior face (the N-terminal residue is in green dotted spheres and the C-terminal residue is in red dotted spheres). Panels B1–4 show residues under selection in the tear ABPBGs. Panels B1 and B3 are exterior views and panels B2 and B4 are interior views. Residues in red are selected with a BEB posterior probability of 99%; those in green with BEB posterior probabilities of 95%; and those in blue with a BEB posterior probability of 90% (the N-terminal ½-Cys residue is a yellow dotted sphere; the C-terminal residue is magenta).

Phyre2 threading predicts that the BG10(15/17) structure is a four-helix bundle with the general boomerang shape of ABP and a fifth helix (**Fig. 2**, Panels A1–2) typical of ABPBG subunits [43], [44]. However, the conserved residues involved in ligand binding and subunit coordination in the dimer [43] are shifted compared with BG2 (shown in **Fig. 2**, Panels B1–2). The secreted BG10(15/17) protein lacks all three Cys residues predicted for ABP subunits (**Fig. 2A**) since a CT/TG substitution following the last codon of the signal peptide caused loss of the first Cys and a frameshift eliminated the remaining two. Thus, the BG10(15/17) subunit cannot dimerize with an ABPA subunit in the traditional, antiparallel, disulfide-bridged form. The *bg10(15/17)* paralogs are in the most recent duplication of the *Abp* gene region, and this region has been shown to vary in copy number in different mouse strains [6]. It is possible that this duplication happened so recently that the involved gene(s) have yet to be down-regulated, although we cannot rule out the possibility that the protein has assumed a novel function (neofunctionalization).

### Quantitative analysis of lacrimal and salivary gland *Abp* transcripts

**Figure 3:**
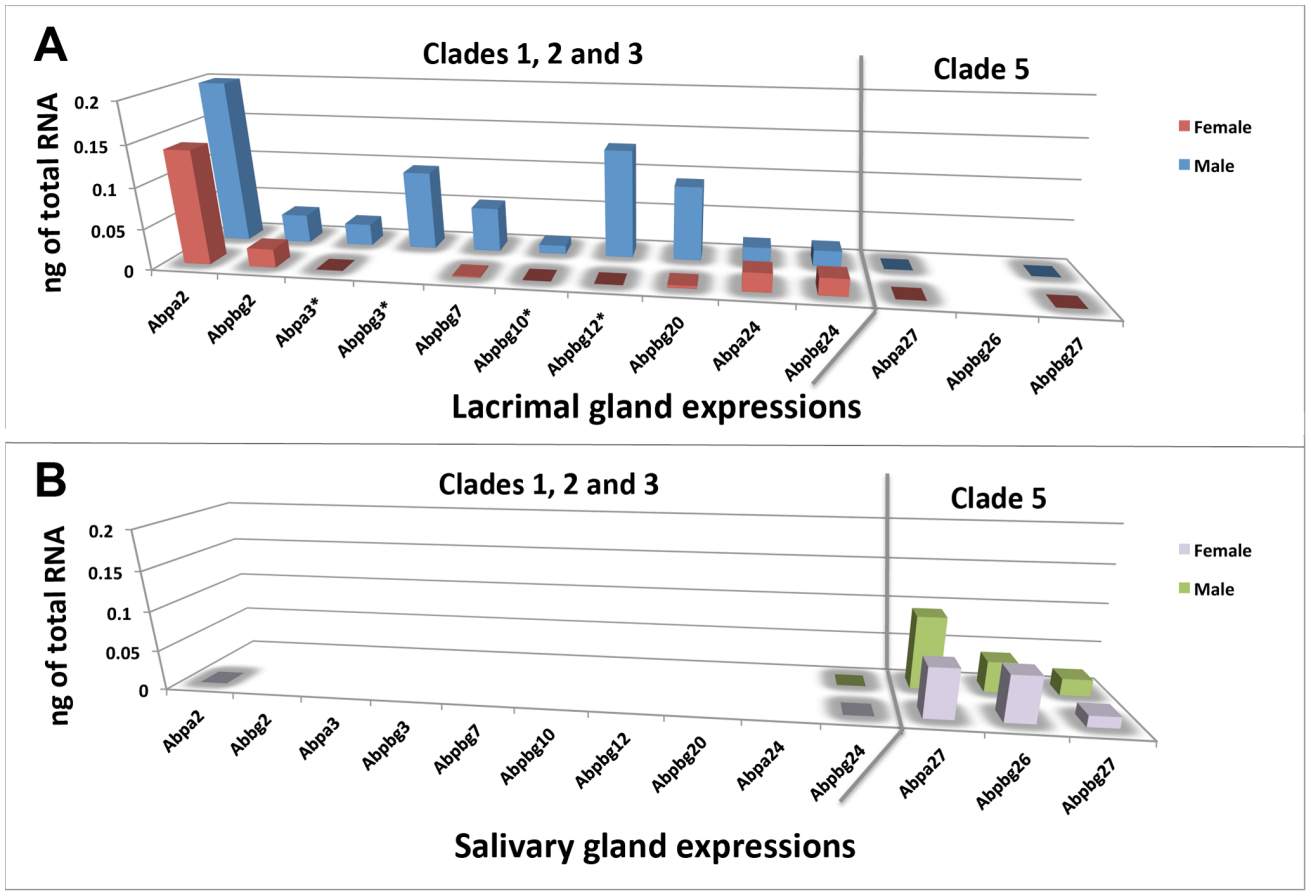
A comparison of *Abp* transcript levels in mouse lacrimal (Panel A) and salivary (Panel B) glands. Expression levels were determined by qPCR (**S9 File in** **S1 Appendix**) and these values are compared with female values in front and male values behind. Paralogs *a12* and *a20* were excluded because of multiple banding. Color legends appear to the right of each graph; the grey line demarcates paralogs found in Clades 1–3 and Clade 5. Paralogs with male sex-limited expression are marked with asterisks. Those paralogs shown by white space are undetectable by qPCR (this varies from the sequence-based identifications in **Fig. 1**).

The *Abp* paralogs *a24*, *a27*, *bg26* and *bg27* are notable because their transcripts are produced in lacrimal glands of both sexes but there are no corresponding proteins in tears. Three of these paralogs (*a27*, *bg26* and *bg27*) are highly expressed in salivary glands and their protein products have been confirmed in the saliva proteomes of both sexes [25]–[27]. The presence of *Abp* transcripts in tears without corresponding peptides in the proteome could be explained if the transcript was present below the threshold of detectable translation product. Because it is based on DNA amplification, PCR can detect a single transcript, while LC-MS/MS does not amplify the level of peptide in a sample. Therefore, we measured transcript levels with qPCR (**S9 File in** **S1 Appendix**) and plot the results in **Fig. 3**. The *a27, bg26* and *bg27* transcripts that lack corresponding tear proteins have 10^5^ less transcript than those paralogs with proteins in tears. This was not true of the *a24* transcript, which was measured at levels in the range of translated transcripts. It is unclear why ABPA24 peptides were not detected in any of the 2-D gel segments.

Similarly, we probed submandibular gland cDNA libraries for *Abp* transcripts without corresponding proteins in a mouse salivary proteome [25], [26]. Non-quantitative PCR from submandibular cDNA libraries identified *a2* and *bg24* transcripts in both sexes of C57BL/6 (**Fig. 1**). Quantitative PCR suggested that these were present at very low levels (**Fig. 3**).

Taking the position that we must be able to identify both a transcript in an exocrine gland and its corresponding protein in the glandular secretion to conclude that an *Abp* paralog is expressed, we conclude that: a) the three *Abp* paralogs found at high levels in mouse saliva have transcripts at very low level in lacrimal glands, but no detectable proteins in tears; b) all the ABP proteins represented by peptides in the LC-MS/MS analyses have transcripts in the glands that produce them.

### Identification of *Abp* genes expressed in lacrimal glands

Our work provides a number of important advances: 1) it increases the number of *Abp* paralogs known to be expressed in lacrimal gland and secreted into tears by adding six paralogs (*bg3, bg7, bg10, a12, bg12* and *bg20*) from the central *Abp* gene region, aka ancestral clade 2 [8]; 2) it corrects the misconception that *a24, a27* and *bg27* (formerly *a8, a11* and *bg11*) are expressed in lacrimal glands [22] since corresponding proteins are not detectable in tears; and 3) it shows that nearly half (5/11; 45%) of lacrimal *Abp* paralogs (*a3*, *a12, bg3*, *bg12* and *bg10*[*15/17*]) are sex-limited in their expression as transcripts in lacrimal glands and proteins in tears of male mice. This entirely male-biased sex-limited expression pattern in lacrimal gland/tears has not been reported previously.

Five of the paralogs expressed as transcripts in lacrimal glands had been previously identified in postnatal animals [22]. Both *a2* and *bg2* (nomenclature unchanged) were expressed from postnatal day 6 to adulthood in both sexes, as were *a24* and *bg24* (previously *a8* and *bg8* [22]). By contrast, *bg3* (previously unpaired *bg12* [9]) was present in both sexes at postnatal days 6 and 15 but only in adult males [22], consistent with the sex-limited expressions we observed in lacrimal glands of adult males. This suggests that the gene is expressed in both sexes before puberty but expression becomes limited to males following puberty.

Lacrimal transcript data from the work of others [24], [28] can be found on NEIbank, which currently reports *Abp* paralogs *a24*, *bg2* and *bg24* as expressed in lacrimal glands based on cloned transcript analysis, as well as *a2*, *a24* and *bg24* in the whole eye. They also report 105 clones as belonging either to *bg7* or to *bg20,* but we have identified representation from *bg15/17* and others in their list using a comparison to our mm9 genome sequences [8]. There is no mention of sex-limited expression of *Abp* genes on NEIbank.

### Positive selection in the evolution of *Abp* paralogs

It has been suggested that selection shaped the evolution of mouse salivary ABP function and promoted the duplication of *Abp* genes (reviewed in [16]). We wished to test whether the *Abpa* and *Abpbg* genes expressed in lacrimal glands and found in tears evolved under different selection regimens from those genes that were not found to be expressed. To do this, we evaluated positive selection on the secreted protein sequences using their *Ka/Ks* ratios (**S10 File in** **S1 Appendix**) and on amino acid sites in the encoded proteins using CODEML (**Tables 2** and **3**). Genetic variation found in sequences results from independent lineage-specific substitutions and substitutions inherited from a common ancestor. Comparisons of *Ka/Ks* ratios across paralogs generated by different duplication events only account for linage specific substitutions if performed in a phylogenetic context as we have here [8]. Moreover, the massive expansion in the genus *Mus* apparently occurred from a single pair of *Abpa* and *Abpbg* genes, minimizing the effect of substitutions inherited from a common ancestor while making identification of orthology with these genes in other mammals impossible [10], [16]. Most importantly, the sets of lacrimal-expressed and non-expressed paralogs we compare are almost entirely from clade 3. This clade has the most recent linage-specific substitutions and therefore our data are not biased by substitutions inherited from a common ancestor.

**Table 2:** Results of the CODEML sites selection test.

Gene Group	Ratio of *dN/dS* (% codons)^1^	*P* Value All Paralogs^2^	Codon Sites Under Selection^3^
Expressed *Abp*as^4^	1 (46%)	*P = *1	None
Non-expressed *Abpa*s^5^	1.8 (20%)	*P = *0.54	None
Expressed *Abpbg*s^6^	5 (24%)	*P = *0.0006	I2, L9, **A24**, **E44**, A50, T52, S69, **T72**, **K73**, **N78**, **K82**
Non-expressed *Abpbg*s^7^	9.6 (1%)	*P = *0.36	K74

1 The *dN/dS* ratio of the class of codons under positive selection is given with the percentage of codon sites predicted to be in that class.

2 The *P*-value rejecting the model of neutral evolution (M8A) over that of selection (M8) is given.

3 Sites with posterior probabilities>0.9 are in regular typeface; sites with *P*>0.95 are in bold typeface and sites with *P*>0.99 in bold, underlined typeface.

4 a12, a20, a3, a2.

5 a15, a17, a24, a19, a7, a11.

6 bg2, bg3p, bg24, bg20, bg7, bg12.

7 bg11, bg18, bg19, bg21, bg1.

**Table 3:** Mouse *Abpbg2* and *Abpbg10* genes^1^ used to produce molecular models.

Paralog	Accession number	Chromosomal Location (strand)^1^	Threaded structure	(results)^2^^,^ 3
*Abpbg2*	KM014055	7: 31302663–31304933 (+)	c2ejnB	(100%; 68; 38%)
*Abpbg10*	KM014058	7: 32092027–32094058 (+)	c2ejnB	(100%; 62; 24%)

1 The protein sequence without signal peptide was threaded for this study.

2 mm10 coordinates.

3 % confidence; length and % identity.

Prior to conducting the *Ka/Ks* analysis, the signal peptide sequences were removed because they are expected to be under a different selective pressure than the regions encoding secreted proteins [45]. Paralogs *bg10*, *bg15*, and *bg17* were also excluded from these analyses because of significant disruptions in their coding regions caused primarily by frame shifts, a conservative change for the purposes of this analysis.

We compared the pairwise *Ka/Ks* ratios (sometimes reported as *dN/dS*; [46]) of ten paralogs expressed in lacrimal gland/tears with the pairwise *Ka/Ks* ratios of fourteen apparently intact genes not expressed in lacrimal gland/tears or salivary glands/saliva. The *Ka*/*Ks* values for unexpressed *Abpbg* paralogs fell near a line of slope 0.5 (**S10 File in** **S1 Appendix**), while the *Ka*/*Ks* values for expressed *Abpbg* paralogs fell between the lines of slope 0.5 and 1.0. The line with slope 1.0 is usually considered to represent neutral variation with respect to selection, however, the averaging effect of *Ka/Ks* computed over all amino acid sites may result in a value less than 1.0 for a protein with a portion of sites under selection. Thus, proteins with *Ka/Ks* values between 0.5 and 1.0 might still be under positive selection [47], [48]. For that reason, we also performed CODEML on *Abp* paralogs.

There was no statistically significant difference for either the expressed or unexpressed *Abpa* groups (*P* = 1 and 0.54 respectively) between the two models (M8 v M8A) indicating that there is no evidence of positive selective pressure acting across these gene phylogenies. The *P* value for the expressed *Abpbg* genes was significant (*P* = 0.0006), while that for the unexpressed *Abpbg* genes was not (*P* = 0.36). The expressed ABPBGs also had eleven amino acid sites identified with high Bayes Empirical Bayes (BEB) posterior probabilities of having evolved under positive selection (*P* = 0.9 to 0.99; **Table 2**). There was only one site with a BEB posterior probability≥0.9 in the unexpressed group. We conclude that the ABPBGs expressed in tears evolved under positive selection, while there is insufficient evidence that the ABPAs did.

The sites in each protein shown to be under selection were mapped on the BG2 structure (**Table 3**). **Fig. 2**, Panels B1–4 show as colored spheres the residues under selection in the ABPBGs expressed in lacrimal glands. Most selected sites in the expressed ABPBGs fall on the exposed subunit face (**Fig. 2**, B3–4), suggesting that these are important for interaction with another molecule(s), e.g. a receptor.

### Selection's influence on the evolution of *Abp* genes expressed in saliva and tears

**Figure 4:**
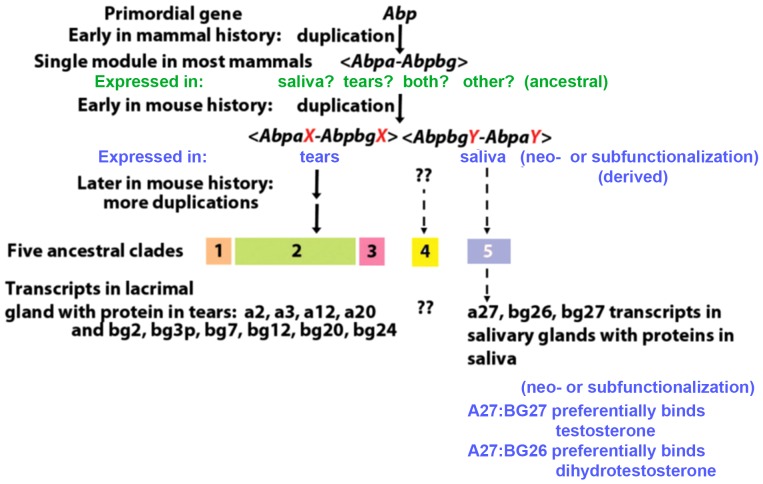
A model for the evolution of *Abp* paralog expression differences in lacrimal gland/tears and salivary gland/saliva. The steps in this evolution are described in the discussion.

In early studies of the *Abp* duplication, we suggested that the dramatic *Abp* expansion in the mouse genome involved two phases with different duplication mechanisms [6]. The initial phase most likely involved duplication of a single ancestral *Abp* gene to produce two paralogs in inverse adjacent order as predicted by Katju and Lynch ([49]; see top of **Fig. 4**). This created the original <*Abpa*-*Abpbg*> module, consistent with the most widespread *Abp* gene configuration in mammal genomes today [8]. Subsequently, this first module probably also underwent simple duplication in the ancestor of the genus *Mus*, producing the forerunners of the first two ancestral clades. These clades, now clades 1 and 5, are in inverse orientation to each other [6] and are represented as modules <*AbpaX-AbpbgX*> and <*AbpbgY-AbpaY*> in **Fig. 4**. The second phase of the expansion involved more rapid *Abp* gene duplication by NAHR, sometimes including unpaired *Abpa* and *Abpbg* paralogs [6]. A breakpoint in a LINE1 retrotransposon, *L1Md_T*, produced during meiotic NAHR creating the most recent duplication in the center of the *Abp* gene region, has been identified [17] (reviewed in [16]). These studies led directly to the evaluation of expression in lacrimal gland and tears that we report here.

A comparison of *Abp* expression in tears and saliva provides some striking conclusions. Considering the five ancestral *Abp* clades [8], all paralogs with transcripts in lacrimal gland and proteins in tears are from clades 1-3 that lie on the proximal side of the *Abp* region (**Fig. 1** and **Fig. 4**). By contrast, the paralogs expressed in submandibular gland and saliva are from clade 5, which lies distal to the other four clades. Thus, based on our criterion of identifying both a transcript in a gland and a protein in its secretion, we conclude that there is no overlap between the paralogs expressed in salivary and lacrimal glands and the clades of their origin (**Fig. 1**). We propose that partitioning of *Abp* expressions occurred early in the evolution of the ancestral clades in the genome of the ancestor of the genus *Mus*.

We have never encountered an ABP subunit that was not involved in a dimer as an alpha disulfide-bridged to a betagamma and so a minimum of one of each gene is apparently required to be expressed in each tissue. This may explain why the <*Abpa*-*Abpbg*> module appears to have been the unit of duplication in the mouse genome and why there are so few unpaired subunit genes.

As **Fig. 4** shows, we do not know whether the single original module was expressed both in lacrimal gland/tears and salivary glands/saliva, or in only one of those (a condition ancestral to the ensuing duplication and neo/subfunctionalization). What seems clear is that duplication would have been required to partition different sets of alpha and betagamma expressions into lacrimal gland/tears on the one hand and salivary glands/saliva on the other (derived characteristics).

This frames the important issue of how selection might have shaped the products of gene family expansion. Theoretical work suggests that neofunctionalization and subfunctionalization are potential fates of daughter gene copies [50]–[54]. We propose that neo/subfunctionalization was responsible for partitioning the expression of eleven *Abp* paralogs from ancestral clades 1, 2 and 3 into lacrimal gland/tears, and three *Abp* paralogs from ancestral clade 5 into submandibular gland/saliva. It seems that the most parsimonious point for this would be when the first <*Abpa*-*Abpbg*> module duplicated to produce modules <*AbpaX-AbpbgX*> and <*AbpbgY-AbpaY*> (**Fig. 4**). While this could have happened later in the region's expansion, it would have required multiple events and is thus less parsimonious.

We observed a number of paralogs transcribed at extremely low levels and without corresponding protein (“bleed through transcription”) in tears or saliva (**Fig. 1**). We argue that this represents a derived condition wherein genes that may have been expressed highly in both glands ancestrally were down-regulated subsequent to duplication as the result of subfunctionalization (**Fig. 4**).

Finally, the partitioning of the two expressions into different ancestral clades could have been stabilized by an accompanying positive selection on multiple amino acid sites in the ABP subunit products, perhaps some of those we show here for the expressed versus non-expressed *Abpbg* genes. It seems to us that the footprints of neo/subfunctionalization can also be seen in the rapid evolution of the *Abp* genes, *a27*, *bg26* and *bg27* [10], [55] that associate in two dimers, A27:BG26 and A27:BG27, and bind testosterone and dihydrotestosterone with different affinities (**Fig. 4**, bottom; [56]). We suggest that the timing of this would have been subsequent to the partitioning of the expression between lacrimal gland/tears and salivary glands/saliva.

We have addressed the question of how two completely different groups of paralogs may have come to be expressed in two exocrine glands, but why would this happen in the first place? It has been proposed that the general function of secretoglobins is to coat and protect surfaces [57] and this seems a more obvious requirement for the eye than the oral cavity. Ironically, sex-limited expression of *Abp* paralogs is seen in lacrimal gland/tears, not in salivary glands/saliva [26], [58]. This hints at an as-yet-undiscovered function of ABP in tears, most likely one involving communication which may supplement or supplant the potential surface-coating role [57]. For example, cuticular hydrocarbons that protect *Drosophila* against desiccation also may act as pheromones [59].

## Conclusions

Using a tripartite approach involving traditional PCR, quantitative PCR and LC-MS/MS proteome analyses, we have clarified and extended the identification of *Abp* paralogs expressed in the lacrimal gland providing secretions to protect the surface of the eye. Thus, ABP in tears may function in surface coating/protection, a role attributed generally to secretoglobins [57]. Considering that the Great Apes have silenced their only *Abpa/Abpbg* gene pair [8] and have no ABP in their tears, it is striking that a rodent would have more than ten of these genes expressed in the major secretion bathing the eye. Morever, we have observed a previously unknown sex-limited expression of five *Abp* paralogs in lacrimal gland/tears of male mice. Both of these observations suggest that there may be an additional function(s) that likely involves communication. Whatever role ABPs play in tears, however, it has been enhanced in the mouse and replaced or lost in the human.

There is evidence that salivary ABP mediates assortative mate selection based on subspecies recognition [12]–[14], limiting gene exchange between subspecies where they meet (reviewed in [10]) and constituting a system of incipient reinforcement along the European hybrid zone [15]. While that provides a precedent for a communication role for the paralogs, it creates the question of how and why two completely different groups of paralogs came to be expressed in each of the two exocrine glands. Our observation that the paralogs expressed in the lacrimal and salivary glands are found in different ancestral clades may provide important clues to how the partitioning of the expressed paralogs came about. We observed a number of instances of extremely low levels of paralog transcription, without corresponding protein production. We argue that this represents a derived condition wherein genes that may have been expressed highly in both glands ancestrally were down-regulated subsequent to duplication as the result of subfunctionalization. Most of the evidence for neo- and/or subfunctionalization has resulted from comparative studies of closely-related species (e.g. [60]–[62]). By contrast, we report evidence for subfunctionalization that partitioned *Abp* paralog expression between salivary and lacrimal glands within a single species.

## Supporting Information

S1 Appendix Contains the following files: **S1 File.** Primer sets used in this study. **S2 File**. Additional details of Methods. **S3 File**. Guide trees used in the CODEML analyses (see also Table 2). **S4 File**. List of all 64 *Abp* paralogs with GenBank accession numbers and coordinates. **S5 File**. Two-dimensional (2D) gel separations of mouse tear proteins. The first dimension was isoelectric focusing (IEF) and the second dimension was SDS gel electrophoresis (SDS). A separation of male tear proteins is shown at the top and a separation of female tear proteins is shown at the bottom. The four red rectangles in each gel cover the likely IEF and SDS ranges and the top and bottom flanking pieces marked M5 (or F5) and M6 (or F6) covered the remaining protein staining regions. All six pieces of each gel were excised and analyzed in LC-MS/MS. **S6 File**. Coverage of transcripts and peptides used to identify ABPA subunits in mouse tears. Also shown are *a24* and *a27* transcripts lacking protein sequences. Transcript sequences identified from cDNA are translated into protein sequence shown in green lowercase typeface. Residues shaded green are individually diagnostic for the paralog in question; those shaded in red are diagnostic as a haplotype. **S7 File**. Coverage of transcripts and peptides used to identify ABPBG subunits in mouse tears. Also shown are *bg26* and *bg27* transcripts lacking protein sequences. Transcript sequences identified in PCR from cDNA libraries are translated into protein sequence shown in green lowercase typeface. Residues shaded green are individually diagnostic for the paralog in question; those shaded in red are diagnostic as a haplotype. **S8 File**. Putative pseudogenes listed by clade and categorized by their changes. Four paralogs (*bg3, bg10, bg15* and *bg17*) are highlighted in green because we identify transcript and protein sequences. **S9 File**. Data from qPCR analyses. **S10 File**. *Ka/Ks* analysis of *Abpa* and *Abpbg* transcript coding regions.
(PDF)
