# Supplementary material for: Did Androgen-Binding Protein Paralogs Undergo Neo- and/or Subfunctionalization as the Abp Gene Region Expanded in the Mouse Genome?
Source: PLoS One. 2014 Dec 22;9(12):e115454. doi: 10.1371/journal.pone.0115454 (PMC4274081; doi:10.1371/journal.pone.0115454)
Supplement: S1 Appendix — Contains the following files: S1 File. Primer sets used in this study. S2 File. Additional details of Methods. S3 File. Guide trees used in the CODEML analyses (see also Table 2). S4 File. List of all 64 Abp paralogs with GenBank accession numbers and coordinates. S5 File. Two-dimensional (2D) gel separations of mouse tear proteins. The first dimension was isoelectric focusing (IEF) and the second dimension was SDS gel electrophoresis (SDS). A separation of male tear proteins is shown at the top and a separation of female tear proteins is shown at the bottom. The four red rectangles in each gel cover the likely IEF and SDS ranges and the top and bottom flanking pieces marked M5 (or F5) and M6 (or F6) covered the remaining protein staining regions. All six pieces of each gel were excised and analyzed in LC-MS/MS. S6 File. Coverage of transcripts and peptides used to identify ABPA subunits in mouse tears. Also shown are a24 and a27 transcripts lacking protein sequences. Transcript sequences identified from cDNA are translated into protein sequence shown in green lowercase typeface. Residues shaded green are individually diagnostic for the paralog in question; those shaded in red are diagnostic as a haplotype. S7 File. Coverage of transcripts and peptides used to identify ABPBG subunits in mouse tears. Also shown are bg26 and bg27 transcripts lacking protein sequences. Transcript sequences identified in PCR from cDNA libraries are translated into protein sequence shown in green lowercase typeface. Residues shaded green are individually diagnostic for the paralog in question; those shaded in red are diagnostic as a haplotype. S8 File. Putative pseudogenes listed by clade and categorized by their changes. Four paralogs (bg3, bg10, bg15 and bg17) are highlighted in green because we identify transcript and protein sequences. S9 File. Data from qPCR analyses. S10 File. Ka/Ks analysis of Abpa and Abpbg transcript coding regions. (PDF) [file pone.0115454.s001.pdf]

File S1. Primer sets used in this study

Paralog	Fwd primer	Primer set	RC primer (5'-3')	Primer set	genomic size	Specific in genomic DNA?
a1p	CATGATGTCCAACTATTTCTTTATG	2004F	GCAAGTAACCAGGCAAGTGGC	2004R	1027 bp	Y
a2 (Abph)	CAGAAGAGTATGTTAATTATGTGGAG	2008F	AAAGATGTAGCCATCAACATAACGG	2008R	934 bp	Y
a3	CTGCAAAAATCAAGCAATGTG	2009q2 F	TAGCGGGCTGGCTTCTATT	2009q2 R	876 bp	N
a4p	GCTTGCTGGTGCTGTGGTTC	2008F	CTTAGACACCTGGAGGGAAGGGA	2008R	1269 bp	-
a5p	GCTGTGGTGATTCTAGGTGCT	2009q2 F	CTGGGCAAATGGCACAAT	2009q2 R	184 bp	N
a6p	TTGCCCAGCTATAAAAGAGGATA	2008F	GGCTTCTATTTTTTCCTGCGT	2008R	981 bp	Y
a7_29	ACCTTGACAGAGGAAGACAAGATAC	2008q1 F	CAAAGTTGTAGCCATCAACATAGC	2008q1 R	867 bp	Y
a8_13p	TCCTCGGGGCTGCCCTGT	2008F	CATAGTCTTATGAAAAAGAATAATAC	2008R	1235 bp	-
a9_14_16p	CAGCTATAAAAGAGGATGTTTGTCTC	2008q1 F	CCATCGACATAGCAGCCTGT	2008q1 R	992 bp	Y
a10_15_17	CCTGCTCCTCCTCTCTTCAT	2008F	AAATGGCTTCCTTGAGAGCAG	2008R	1176 bp	Y
a11_18	GGTTGTTATTACCGTGTGGGT		CACCTGGAGAGAAGGACTGT	2008R	1200 bp	Y
a12	GAAACAATACAAAGATGACCCTGTAAC	2008q1 F	CCTAGAGGGAAAGACTATAATCTGCC	2013R	1002 bp	Y
a19	ATGAAGCTTGCTGGTGCTG	2013F	CATAGTGGGCTGGCTTCTATC	2013R	1228 bp	N
a20	ACCTTGACAGAGGAAGACAAGATAC	2008q1 F	CCAAAATTGTAGCCATCAACATATC	2008q1 R	873 bp	Y
a21p	AGGATGTTCACCTATTTTTAAACAG	2008F	GCTGGCTGGCTTCTATTTTTC	2008R	968 bp	Y
a22p	GCTGTGGTGATCCTCAGGC	2008F	TCATCTATGCAGATGAGCAGAA	2008R	1225 bp	Y
a23p	CTGGTGCTTTGATGATCCTTGA	2008F	GTGGCCATCAACATGCCGTG	2008R	1171 bp	Y
a24	GATGTTCATCTATTCTTTCACAGG	2004F	AAGATGCAAACATCAACATGCTGGT	2004R	987 bp	Y
a25p	GCTGCACTGCTCCTGACTG	2008F	TGACTTCCTTCAGAGCTTCCT	2008R	1135 bp	Y
a26p	AAGAGTATGTTGAGTACCTGAAAC	2008F	CTGGTGAGCAGGCAAATGG	2008R	988 bp	Y
a27	ACAGAGAAAAGTTGATTTATTTTTGAATG	2004F	GGAGGCAATTGGTTTCCG	2004R	1019 bp	Y
a28p	GAGGAGGTTCGTCTATTTTTAAACG	2004F	AAGTTAGAGCCATCAACATAGTGT	2004R	981 bp	Y
a30p	AACAATACAATAATGACCCTCTCG	2004F	ACTGGTGAGCAGCTAAGTGGC	2004R	968 bp	Y

Paralog	Fwd primer	Primer set	RC primer (5'-3')	Primer set	genomic size	Specific in genomic DNA?
bg1	GGGTGTCGTCTCTGGATACA	2008F (old bg1)	CAAGACTTCTTTGGTATAATATGACT	2008R (old bg1)	1203 bp	Y
bg2 (Abpe)	TTTGGGTATTCTCTCTGGAAACA	2008F (old bg2)	AGAATGTTCTTCAAGACTTCTTTG	2008R (old bg2)	1213 bp	Y
bg3p	TTGAGCACTGTCTCTGGGAAAA	2008F (old bg12)	CATCCATTTCTTGGTAATCACAC	2008R (old bg12)	1211 bp	Y
bg4p	AAACATACTTCTGGAACGCAAAA	2009q2 F	GCCCCTTCTATAGTGTGACTGG	2009q2 R	1065 bp	N
bg5p	GGGCTGCTATAGAGAGGAAAGAT	2009q2 F	CGGTGTCCCTTCTATAGTGTGC	2009q2 R	1092 bp	N
bg6p	GGACACTTCTTCTGCTGACCTT	2008q1 F	GTCTGGAGTTCTTGATACAATCTTTTC	2008q1 R	594 bp	Y
bg7	GCATGTTTTCCTTTCTTTGAAGC	2013F	CCATAGTATGACAGGCATTCAGG	2009q2 R	1212 bp	Y
bg8_13p	TTTAATGGTTTATAAAGAAGAGGAGCTT	2008q1 F	TCCTCTGTAGCAGCCACAGA	2008q1 R	712 bp	N
bg9_14_16p	GGGCTGCTATAGAGAGGAAAGAT	2009q2 F	CGGTGTCCCTTCTATAGTGTGC	2009q2 R	1103 bp	N
bg10_15_17p	TAAAGGTGGCCTTGGAAAAA	2009q2 F	GCTGGCAATCATGGCTTC	2009q2 R	1089 bp	Y
bg11_18	GGAGAACTGAGCTTCCAGACAT	2008q1 F	CTTGCATAGCCTTCAAAGAAAGA	2008q1 R	523 bp	Y
bg12	ACTCTTCACCATGAAGGGGAT	2013F	GAAATAAAGTCTAAAATGGATCTAAAATTGTC	2013R	1779 bp	Y
bg19	CAGGGTTGCTACAGAGAGGAA	bg12/19 2009q2F	AGGTTGGAAATAAAGTCTAAAATGGA	bg12/19 2009q2F	1125 bp	Y
bg20 (Abpd)	TGTCTTCCTTTCTTCGAAGGC	2013F	CCATAGTATGACAGGCATTCAGG	bg7 2009q2R	1207 bp	Y
bg21	TGCAAGTGTTGTCTCTGGAAGT	2008F (old bg5)	GTCCTAATAGCTTCGAAAGAAGG	2008R (old bg5)	1237 bp	Y
bg22p		-		-	-	-
bg23p		-		-	-	-
bg24 (Abpz)	GCTGGTGTTATCTCAGGAAGCA	2008F (old bg8)	CGTAAAGGTTTCTTCGGAATAGT	2008R (old bg8)	1202 bp	Y
bg25p	GCAGTACTCTCTGGACTTAAGAT	2008F (old bg9)	CCATGTCTTGATAAATAGAAGCC	2008R (old bg9)	didn't show u	Y
bg26	CGGAGCAATACTTACTCTAAGG	2008F (old bg10)	CTTTAAGAGGTCATTGCCATAGT	2008R (old bg10)	1217 bp	Y
bg27	TAAAATACTGGGTGGAAATAGGC	2008F (old bg11)	CCGCCATTTTGTTTACAGAATCT	2008R (old bg11)	1208 bp	Y
bg28	CAAACTTCTGGAACCCAAAATTA	2009q2 F	AGTGCCCCTTTATAGTGTGACTG	2009q2 R	1041 bp	Y
bg29_31_32p	CACTGTGAGGAGCATTTAGCACT	2008q1 F	ATAGCCTTTGAAGTAAGGAACACATT	2008q1 R	588 bp	Y
bg30_33p	GCTACAGAGAGGAAAAATTAAGAAACA	2009q2 F	GGTTGGAAATAAAGTCTAAAATGGAT	2009q2 R	1126 bp	Y
bg34p		-		-	-	-

The symbol "-" means that the primer did not amplify anything
Primer sets dated 2004 were published in ref. 9

File S2. Supplementary Materials and Methods

Mass spectrometry and proteome data were acquired by the Arizona Proteomics Consortium supported by NIEHS grant ES06694 to the SWEHSC, NIH/NCI grant CA023074 to the AZCC and by the BIO5 Institute of the University of Arizona. The Thermo Fisher LTQ Orbitrap Velos mass spectrometer was provided by grant 1S10 RR028868-01 from NIH/NCRR.

Transcript analyses

RNA isolation was performed with a GenElute Total RNA mini-prep kit (Sigma). PCR was performed with Dream Taq (Fermentas) and primers provided by Bioneer. Primer sequences are available from RCK on request. Dye-terminator DNA sequencing was done on Applied Biosystems 3730 DNA Analyzers by the University of Arizona UAGC laboratory using the BigDye® Terminator v3.1 Cycle Sequencing Kit.

Preparation of samples for mass spectrometric analysis and two-dimensional gel separation

Tear fluids were provided to the Proteomics Core Facility of the University of Arizona where personnel determined the protein quantity in the samples using the Pierce 660 nm assay (http://www.piercenet.com/). A total of 175 µg of sample was used. The sample was mixed into a solution with a final concentration of 7 M Urea/2 M Thiourea/ 4% CHAPS. No reducing agent was used. The samples were then introduced to an 11 cm pH 3-10 linear IPG strip (Biorad, Hercules CA) using the Protean IEF Cell (Biorad). The IEF strips were rehydrated and focused using a linear method with a 16_{o} active rehydration. The focusing was considered complete when 35000 v/hours were reached. Electrode Wicks (Biorad) were placed between the strip and the actual electrode wire during the first step of the focusing procedure to absorb excess salts. The focused IEF strips were equilibrated in a solution of 6 M urea/0.375 M Tris/2% SDS/ 20% Glycerol for 15 minutes, no reduction or alkylation was done on the strips. The strips were then individually placed onto their own 12.5% Criterion IPG +1 well gel (Biorad) and overlaid with a 1% agarose solution containing bromophenol blue dye to track the progress of the run. 4 µl of Precision Plus Standard (Biorad) was added to the standard well. The gels were run together on a Criterion gel cell (Biorad) for 15 min at 100 V and then 150V until the tracking dye reached the bottom of

each gel. The gels were then rinsed 3 times with deionized water and then stained with Biorad Bio-Safe Coomassie stain per the manufacturer's instructions (Biorad). The gels were imaged and individual spots or sections of spots were cut out and then digested as described previously with trypsin (Karn & Laukaitis, 2011).

Tandem mass spectrometry coupled to liquid chromatography (LC-MS/MS) of 2D separated proteins

LC-MS/MS analysis of trypsin digested 2D-SDS-PAGE gel pieces (Shevchenko et al., 1996) was carried out using a LTQ Orbitrap Velos mass spectrometer (Thermo Fisher Scientific, San Jose, CA) equipped with an Advion nanomate ESI source (Advion, Ithaca, NY), following ZipTip (Millipore, Billerica, MA) C18 sample clean-up according to the manufacturer's instructions. Peptides were eluted from a C18 precolumn (100-μm id × 2 cm, Thermo Fisher Scientific) onto an analytical column (75-μm ID × 10 cm, C18, Thermo Fisher Scientific) using a 5-10% gradient of solvent B (acetonitrile, 0.1% formic acid) over 5 minutes, followed by a 10-35% gradient of solvent B over 35 minutes, 35-50% gradient of solvent B over 20 minutes, 50-95% gradient of solvent B over 5 minutes, and finally by a 95% solvent B hold for another 5 minutes. Solvent A consisted of water and 0.1% formic acid. Data dependent scanning was performed by the Xcalibur v 2.1.0 software (Andon et al., 2002) using a survey mass scan at 60,000 resolution in the Orbitrap analyzer scanning *m/*z 400-1600, followed by collision-induced dissociation (CID) tandem mass spectrometry (MS/MS) of the fourteen most intense ions in the linear ion trap analyzer. Precursor ions were selected by the monoisotopic precursor selection (MIPS) setting with selection or rejection of ions held to a +/- 10 ppm window. Dynamic exclusion was set to place any selected *m/z* on an exclusion list for 45 seconds after a single MS/MS. All MS/MS spectra were searched against Uniprot *Mus musculus* downloaded January 11, 2012 (http://www.uniprot.org/taxonomy/10090), appended with ABP sequences provided by Robert Karn, using Thermo Proteome Discoverer 1.3 (Thermo Fisher Scientific). At the time of the search, this combined protein database contained 50,565 entries. Proteins were identified at 99% confidence with XCorr score cut-offs (Qian et al., 2005) as determined by a reversed database search. The results were displayed with Scaffold v 3.6.1 (Proteome Software Inc., Portland OR), a program that relies on various search engine results (ie: Sequest,

X!Tandem, MASCOT) and which uses Bayesian statistics to reliably identify more spectra (Keller et al., 2002, Nesvizhskii et al., 2003).

Criteria for protein identification

Scaffold was used to validate MS/MS based peptide and protein identifications. Peptide identifications were accepted if they exceeded specific database search engine thresholds. Sequest identifications required at least deltaCn scores of greater than 0.08 and XCorr scores of greater than 1.8, 2.5, 3.5 for singly, doubly, triply charged peptides. Protein identifications were accepted if they contained at least 2 identified peptides. Proteins that contained similar peptides and could not be differentiated based on MS/MS analysis alone were grouped to satisfy the principles of parsimony.

Data analysis

The program DNAsp was used to obtain the rates of nonsynonymous (*Ka*) and synonymous (*Ks*) nucleotide substitutions (Librado & Rozas, 2009). Mouse *Abp* transcript cDNA sequences were modified to contain only the coding regions for their respective secreted protein products (i.e. signal peptides were removed) and these were aligned using CLUSTALX (Jeanmougin et al., 1998, Thompson et al., 1997).

Phylogenetic trees were constructed from the alignments using the program PAUP* (Swofford, 1998) using neighbor joining (NJ) distance parameters with Jukes-Cantor correction and random-seeding. These phylogenies were used as guide trees for CODEML analysis.

Andon, N. L., Hollingworth, S., Koller, A., Greenland, A. J., Yates, J. R., 3rd & Haynes, P. A. 2002. Proteomic characterization of wheat amyloplasts using identification of proteins by tandem mass spectrometry. *Proteomics* **2**: 1156-68.

Jeanmougin, F., Thompson, J. D., Gouy, M., Higgins, D. G. & Gibson, T. J. 1998. Multiple sequence alignment with Clustal X. *Trends Biochem Sci* **23**: 403-5.

Karn, R. C. & Laukaitis, C. M. 2011. Positive selection shaped the convergent evolution of independently expanded kallikrein subfamilies expressed in mouse and rat saliva proteomes. *PLoS ONE* **6**: e20979.

Keller, A., Nesvizhskii, A. I., Kolker, E. & Aebersold, R. 2002. Empirical statistical model to estimate the accuracy of peptide identifications made by MS/MS and database search. *Anal Chem* **74**: 5383-92.

Librado, P. & Rozas, J. 2009. DnaSP v5: a software for comprehensive analysis of DNA polymorphism data. *Bioinformatics* **25**: 1451-2.

Nesvizhskii, A. I., Keller, A., Kolker, E. & Aebersold, R. 2003. A statistical model for identifying proteins by tandem mass spectrometry. *Anal Chem* **75**: 4646-58.

Qian, W. J., Monroe, M. E., Liu, T., Jacobs, J. M., Anderson, G. A., Shen, Y., Moore, R. J., Anderson, D. J., Zhang, R., Calvano, S. E., Lowry, S. F., Xiao, W., Moldawer, L. L., Davis, R. W., Tompkins, R. G., Camp, D. G., 2nd & Smith, R. D. 2005. Quantitative proteome analysis of human plasma following in vivo lipopolysaccharide administration using 16O/18O labeling and the accurate mass and time tag approach. *Mol Cell Proteomics* **4**: 700-9.

Shevchenko, A., Wilm, M., Vorm, O., Jensen, O. N., Podtelejnikov, A. V., Neubauer, G., Shevchenko, A., Mortensen, P. & Mann, M. 1996. A strategy for identifying gel-separated proteins in sequence databases by MS alone. *Biochem Soc Trans* **24**: 893-6.

Swofford, D. L. 1998. *PAUP*. Phylogenetic Analysis Using Parsimony (*and Other Methods)*. Sinauer Associates, Sunderland, MA.

Thompson, J. D., Gibson, T. J., Plewniak, F., Jeanmougin, F. & Higgins, D. G. 1997. The CLUSTAL_X windows interface: flexible strategies for multiple sequence alignment aided by quality analysis tools. *Nucleic Acids Res* **25**: 4876-82.

File S3. Guide Trees

Expressed Abpa's
1

((1,2),3,4);

// end of file

1: a12
2: a20
3: a3
4: a2

Non-expressed Abpa's
1

((((1,2),3),4),5,6);

// end of file

1: a15
2: a17
3: a24
4: a19
5: a7
6: a11

Expressed Abpbg's
1

(((1,3),2),(4,5),6);

// end of file

1: bg2
2: bg3p
3: bg24
4: bg20
5: bg7
6: bg12

Non-expressed Abpbg's
1

(((1,2),3),4,5);

// end of file

1: bg11
2: bg18
3: bg19
4: bg21
5: bg1

File S4. List of all 64 Abp paralogs with GenBank accession numbers and coordinates

GENE	GenBank Acc #	CHR	STRAND	mm9 coordinates		mm10 coordinates		LENGTH
B6_a1ψ	KM014069	7	n	32039982	32041416	31254963	31256397	1435
**B6_bg1**	KM014083	7	p	32048657	32050908	31263638	31265889	2252
**B6_a2**	KM014043	7	n	32075543	32076939	31290524	31291920	1397
B6_bg2	KM014055	7	p	32087682	32089952	31302663	31304933	2271
B6_bg3ψ	KM014056	7	n	32143937	32147206	31358918	31362187	3270
**B6_a3**	KM014044	7	p	32160507	32161931	31375488	31376912	1425
B6_bg4ψ	KM014084	7	n	32185931	32188316	31400912	31403297	2386
B6_a4ψ	KM014070	7	p	32194574	32195996	31409555	31410977	1423
B6_a28ψ	KM014081	7	p	32228722	32230136	31443703	31445117	1415
B6_bg5ψ	KM014085	7	n	32285081	32286475	31500062	31501456	1395
B6_a5ψ	KM014071	7	p	32292340	32293744	31507321	31508725	1405
B6_bg28ψ	KM014094	7	n	32329941	32331947	31544922	31546928	2007
B6_bg6ψ	KM014086	7	n	32402639	32404664	31617620	31619645	2026
B6_a6ψ	KM014072	7	p	32410499	32411928	31625480	31626909	1430
**B6_bg7**	KM014057	7	n	32488842	32490875	31703823	31705856	2034
**B6_a7**	KM014045	7	p	32497586	32498993	31712567	31713974	1408
B6_bg8ψ	KM014087	7	n	32563678	32565696	31778659	31780677	2019
B6_a8ψ	KM014073	7	p	32573178	32574586	31788159	31789567	1409
B6_bg9ψ	KM014088	7	n	32700132	32702523	31915113	31917504	2392
B6_a9ψ	KM014074	7	p	32709242	32710665	31924223	31925646	1424
B6_bg29ψ	KM014095	7	n	32797931	32799937	32012912	32014918	2007
B6_bg10ψ	KM014058	7	n	32877046	32879077	32092027	32094058	2032
**B6_a10**	KM014046	7	p	32885768	32887186	32100749	32102167	1419
B6_bg30ψ	KM014096	7	n	32931141	32933170	32146122	32148151	2030
**B6_bg11**	KM014059	7	n	32994236	32996260	32209217	32211241	2025
**B6_a11**	KM014047	7	p	33007756	33009172	32222737	32224153	1417
**B6_bg12**	KM014060	7	n	33110343	33112366	32325324	32327347	2024
**B6_a12**	KM014048	7	p	33119097	33120516	32334078	32335497	1420
**B6_a29**	KM014045	7	p	33226445	33227857	32441426	32442838	1413
B6_bg13ψ	KM014089	7	n	33292401	33294418	32507382	32509399	2018
B6_a13ψ	KM014075	7	p	33301910	33303318	32516891	32518299	1409
B6_bg14ψ	KM014090	7	n	33432547	33434939	32647528	32649920	2393
B6_a14ψ	KM014076	7	p	33441664	33443088	32656645	32658069	1425
B6_bg31ψ	KM014097	7	n	33530194	33532200	32745175	32747181	2007
B6_bg15ψ	KM014061	7	n	33612747	33614778	32827728	32829759	2032
**B6_a15**	KM014049	7	p	33621469	33622887	32836450	32837868	1419
B6_bg16ψ	KM014090	7	n	33659437	33661829	32874418	32876810	2393
B6_a16ψ	KM014076	7	p	33668554	33669978	32883535	32884959	1425
B6_bg32ψ	KM014097	7	n	33757093	33759099	32972074	32974080	2007
B6_bg17ψ	KM014061	7	n	33839646	33841677	33054627	33056658	2032
**B6_a17**	KM014049	7	p	33848364	33849782	33063345	33064763	1419
B6_bg33ψ	KM014098	7	n	33896570	33898596	33111551	33113577	2027
**B6_bg18**	KM014062	7	n	33956955	33958979	33171936	33173960	2025
**B6_a18**	KM014047	7	p	33970467	33971883	33185448	33186864	1417

**B6_bg19**	KM014063	7	n	34063433	34065457	33278414	33280438	2025
**B6_a19**	KM014050	7	p	34072206	34073626	33287187	33288607	1421
**B6_bg20**	KM014064	7	n	34149406	34151437	33364387	33366418	2032
**B6_a20**	KM014051	7	p	34158154	34159571	33373135	33374552	1418
B6_bg34ψ	KM014099	7	n	34227925	34229954	33442906	33444935	2030
**B6_bg21**	KM014065	7	n	34303544	34305567	33518525	33520548	2024
B6_a21ψ	KM014077	7	p	34312275	34313697	33527256	33528678	1423
B6_bg22ψ	KM014091	7	n	34389169	34391191	33604150	33606172	2023
B6_a22ψ	KM014078	7	p	34399532	34400942	33614513	33615923	1411
B6_bg23ψ	KM014092	7	n	34410209	34412238	33625190	33627219	2030
B6_a23ψ	KM014079	7	p	34420061	34421471	33635042	33636452	1411
**B6_bg24**	KM014066	7	n	34522246	34524428	33737227	33739409	2183
**B6_a24**	KM014052	7	p	34528700	34530118	33743681	33745099	1419
B6_bg25ψ	KM014093	7	n	34637923	34639956	33852904	33854937	2034
B6_a25ψ	KM014080	7	p	34652853	34654216	33867834	33869197	1364
**B6_bg26**	KM014067	7	n	34728065	34730103	33943046	33945084	2039
B6_a26ψ	KM014053	7	p	34743704	34745121	33958685	33960102	1418
**B6_bg27**	KM014068	7	n	34796983	34799005	34011964	34013986	2023
**B6_a27**	KM014054	7	p	34806467	34807888	34021448	34022869	1422
B6_a30ψ	KM014082	7	p	34884647	34885844	34099628	34100825	1198

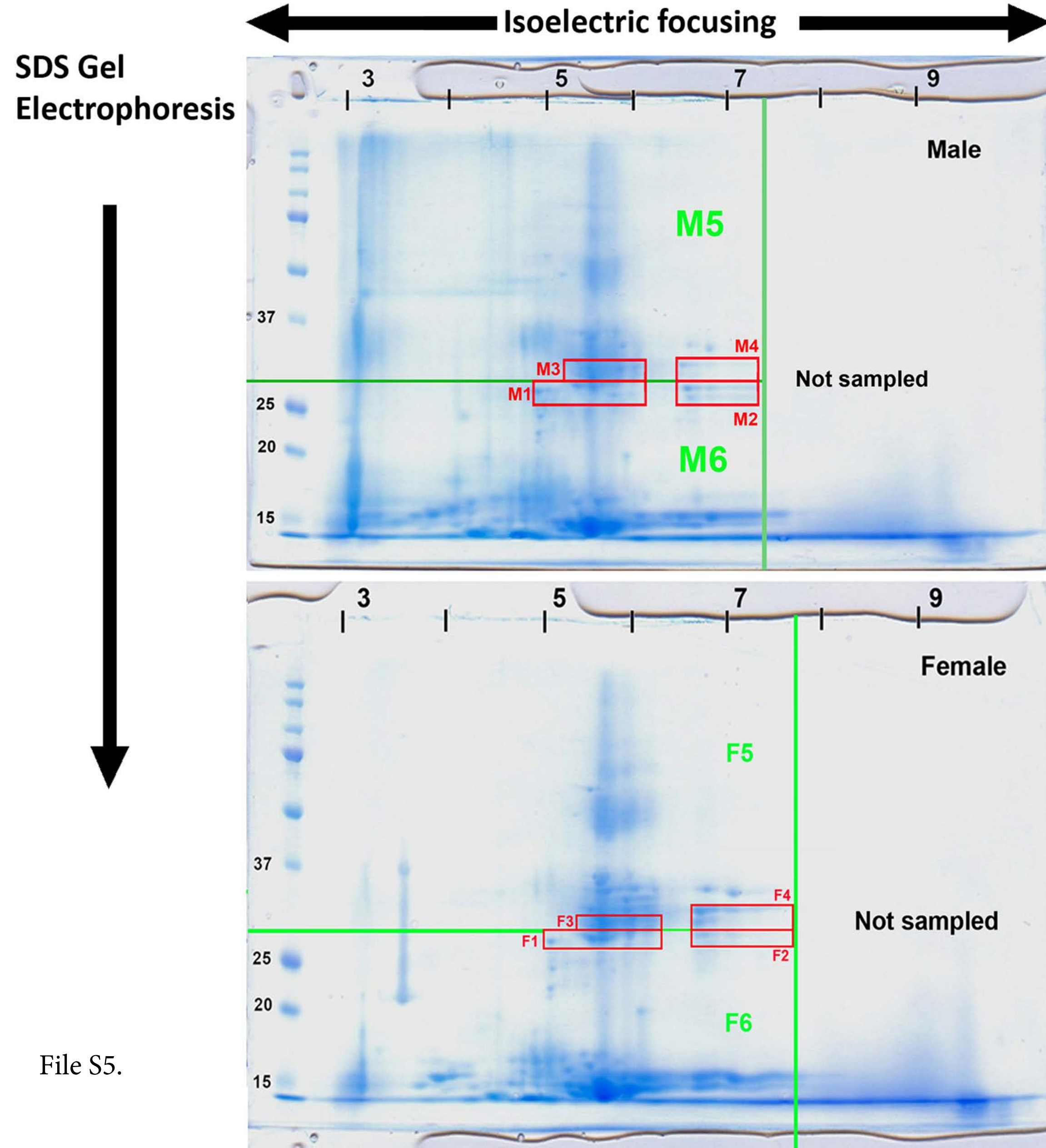
Isoelectric focusing
SDS Gel
Electrophoresis
3
5
7
9
Male
M5
37
M4
M3
Not sampled
M1
25
M2
20
M6
15
3
5
7
9
Female
F5
37
F4
F3
Not sampled
F1
25
F2
20
F6
15

File S5.

File S6.

```
a2
gicpaikedvhlflfgipeeyvnyvekykddpetlenteklkicvdrtltkenkehaaafi              M & F transcript
        hlflfgipeeyvnyvekykddpetlenteklkicvdrtltkenkehaaafiekiessplc
GICPAIKedvhlflfgtpeeyvnyvekYKDDPETLENTEKLKicvdrTLTKENKEHAAAFIEKIESSPLC     M1 (gel piece)
gicpaikedvhlflfgtpeeyvnyvekYKDDPETLENTEKlkicvdrtltkenkehaaafiekIESSPLC     M2   "   "
GICPAIKEDVHLFLFGTPEEYVNYVEKYKDDPETLENTEKLKicvdrTLTKENKEHAAAFIEKIESSPLC     M3   "   "
gicpaikEDVHLFLFGTPEEYVNYVEKYKDDPETLENTEKlkicvdrtltkENKEHAAAFIEKIESSPLC     M4   "   "
gicpaikedvhlfLFGTPEEYVNYVEKYKDDPETLENTEKlkicvdrtltkENKEHAAAFIEKIESSPLC     M5   "   "
GICPAIKEDVHLFLFGTPEEYVNYVEKYKDDPETLENTEKlkicvdrTLTKENKEHAAAFIEKIESSPLC     M6   "   "
GICPAIKEDVHLFLFGTPEEYVNYVEKYKDDPETLENTEKlkicvdrtltkENKEHAAAFIEKIESSPLC     F1   "   "
gicpaikedvhlflfgtpeeyvnyvekykddpetlenteklkicvdrtltkENKEHAAAFIEKIESSPLC     F2   "   "
GICPAIKEDVHLFLFGTPEEYVNYVEKYKDDPETLENTEKLKicvdrtltkENKEHAAAFIEKIESSPLC     F3   "   "
gicpaikedvhlflfgtpeeyvnyvekYKDDPETLENTEKlkicvdrtltkENKEHAAAFIEKiessplc     F4   "   "

a3
gicpaikedvrlflngtseeyveyvkqykddpeilentakikqcvds                            M transcript
GICPAIKEDVRLFLNGTSEEYVEYVKQYKDDPEILENTAKIKQCVDSTLTEEDKAHATAFIEKieasplc     M1 (gel piece)
GICPAIKEDVRlflngtseeyveyvkQYKDDPEILENTAKikqcvdstlteedkahatafiekieasplc     M3   "   "
GICPAIKEDVRlflngtseeyveyvkqykddpeilentakIKQCVDSTLTEEDKAHATAFIEKieasplc     M4   "   "
GICPAIKEDVRLFLNGTSEEYVEYVKQYKDDPEILENTAKIKQCVDSTLTEEDKAHATAFIEKieasplc     M5   "   "
GICPAIKEDVRLFLNGTSEEYVEYVKQYKDDPEILENTAKIKQCVDSTLTEEDKAHATAFIEKIEASPLC     M6   "   "

    m in a12
mklagamvilgaallllltsggdc              t in 12     d in 12              M transcript
    v in a19                          v in 19     n in 19

a12
gicpaikedvrlflngtseayveyvkqykddpvtlentakikqcvdstlteedrahattfiekieasplc     M transcript
                                                                             [continued]
GICPAIKEDVRlflngtseayveyvkqykddpvtlentakIKQCVdSTLTEedrAHATTFIEKieasplc     M5 (gel piece)

a20
gicpaikedvrlflngtseeyveyvkqykddpvilentakikqcvdstlteedkihattfiedieaspic     M & F transcript
GICPAIKEDVRLFLNGTSEEYVEYVKQYKDDPVILENTAKIKQCVDSTLTEEDKIHATTFIEKIEASPIC     M1 (gel piece)
gicpaikedvrlflngtseeyveyvkqykddpvilentakikqcvdstlteedkihattfiekIEASPIC     M2   "   "
GICPAIKEDVRlflngtseeyveyvkQYKDDPVILENTAKIKQCVDSTLTEEDKIHATTFIEKieaspic     M3   "   "
GICPAIKEDVRlflngtseeyveyvkQYKDDPVILENTAKIKQCVDSTLTEEDKIHATTFIEKIEASPIC     M4   "   "
GICPAIKEDVRLFLNGTSEEYVEYVKQYKDDPVILENTAKIKQCVDSTLTEEDKIHATTFIEKIEASPIC     M5   "   "
GICPAIKEDVRLFLNGTSEEYVEYVKQYKDDPVILENTAKIKQCVDSTLTEEDKIHATTFIEKIEASPIC     M6   "   "
GICPAIKEDVRlflngtseeyveyvkQYKDDPVILENTAKIKQCVDSTLTEEDKIHATTFIEKIEASPIC     F1   "   "
GICPAIKedvrlflngtseeyveyvkQYKDDPVILENTAKIKQCVDSTLTEEDKIHATTFIEKieaspic     F3   "   "
                             not a7 because of the ATA codon for I

a24
      vhlffhrtseeyveyvkqykddpeilentekikkcvdstltdedkthatafiekiearpac        M & F transcript
a27
    qrkvdlflngtteeyveylkqfnentkvlenaanikkcsdrtlteedkaqatslinkitasrtc       M & F transcript
```

File S7.

```
bg2
   lgilsgnriglhtelapfdptveekeafekiqdcyeeeglkaktedmklmttilfssecrsyytkevlknilvkfskklt M/F transcript
cipffgvylgilsgnrIGLHTELAPFDPTVEEKEAFEKIQDCYEEEGLKAKTEDMKLMTTILFSSECRSYYTKEVLKNILVKfskklt M1 gel piece
cipffgvylgilsgnrIGLHTELAPFDPTVEEKEAFEKIQDCYEEEGLKAKTEDMKLMTTILFSSECRSYYTKEVLKNILVKfskklt M2 "  "
cipffgvylgilsgnrIGLHTELAPFDPTVEEKEAFEKIQDCYEEEGLKAKTEDMKLMTTILFSSECRSYYTKEVLKNILVKfskklt M3 "  "
cipffgvylgilsgnrIGLHTELAPFDPTVEEKEAFEKIQDCYEEEGLKAKTEDMKLMTTILFSSECRsyytkevlknilvkfskklt M4 "  "
cipffGVYLGILSGNRIGLHTELAPFDPTVEEKEAFEKIQDCYEEEGLKAKTEDMKLMTTILFSSECRsyytkevlknilvkfskklt M5 "  "
CIPFFGVYLGILSGNRIGLHTELAPFDPTVEEKEAFEKIQDCYEEEGLKAKTEDMKLMTTILFSSECRSYYTKEVLKNILVKfskklt M6 "  "
CIPFFGVYLGILSGNRIGLHTELAPFDPTVEEKEAFEKIQDCYEEEGLKAKTEDMKLMTTILFSSECRsyytkevlknilvkfskklt F1 "  "
cipffgvylgilsgnrIGLHTELAPFDPTVEEKEAFEKIQDCYEEEGLKAKTEDMKLMTTILFSSECRsyytkevlknilvkfskklt F2 "  "
CIPFFGVYLGILSGNRIGLHTELAPFDPTVEEKEAFEKIQDCYEEEGLKAKTEDMKLMTTILFSSECRSYYTKEVLKNILVKfskklt F3 "  "
cipffgvylgilsgnrIGLHTELAPFDPTVEEKEAFEKIQDCYEEEGLKaktedmkLMTTILFSSECRsyytkevlknilvkfskklt F4 "  "
cipffgvylgilsgnrIGLHTELAPFDPTVEEKEAFEKIQDCYEEEGLKAKTEDMKLMTTILFSSECRsyytkEVLKNILVKfskklt F5 "  "
cipffgvylgilsgnrIGLHTELAPFDPTVEEKEAFEKIQDCYEEEGLKAKTEDMKLMTTILFSSECRSYYTKEVLKNILVKfskklt F6 "  "

bg3
   lstvsgkrlwlhhelsyfnptdgetksfkkiqdcyeeaglkaksqdvqfmasmffsseclkyysndtmtkilsvitkkwm M transcript
casffgvylstvsgkrlwlhhelsyfnptdgetksfkkiqdcyeeaglkaksqdvqfmasmffsseclkyysndtmtkILSVITKKwm M1 gel piece
casffgvylstvsgkrlwlhhelsyfnptdgetksfkkiqdcyeeaglkaksqdvqfmasmffsseclkyysndtmtkILSVITKKwm M2 "  "
casffgvylstvsgkrlwlhhelsyfnptdgetksfkKIQDCYEEAGLKaksqdvqfmasmffsseclkyysndtmtkILSVITKKwm M3 "  "
casffgvylstvsgkrlwlhhelsyfnptdgetksfkKIQDCYEEAGLKaksqdvqfmasmffsseclkyysndtmtkILSVITKKwm M5 "  "
casffgvylstvsgkRLWLHHELSYFNPTDGETKsfkKIQDCYEEAGLKakSQDVQFMASMFFSSECLKYYSNDTMTKILSVITKKwm M6 "  "

                                n(20/29)                r(20)
 l(20)     v(29)  m(29)                         (29 disrupted from here)
                                   *                      *
bg7
acfpffeayasvlsgsrvwlyqelqafdataeekvalekiqdcyseesirnillepkimeamvaspeclsyyg  M & F transcript
cfpffeayasvlsgsrvwlyqelqafdataeekvalekIQDCYSEESIRNILLEPKIMEAMVASPECLSYYGLDNIRSILDYISKllge M1 gel piece
cfpffeayasvlsgsrvwlyqelqafdataeekvalekIQDCYSEESIRnillepkimeamvaspeclsyygldnirSILDYISKllge M2 "  "
cfpffeayasvlsgsrvwlyqelqafdataeekvalekIQDCYSEESIRNILLEPKimeamvaspeclsyygldnirSILDYISKllge M3 "  "
cfpffeayasvlsgsrVWLYQELQAFDATAEEKvalekIQDCYSEESIRnillepkIMEAMVASPECLSYYGLDNIRSILDYISKllge M4 "  "
cfpffeayASVLSGSRvwlYQELQAFDATAEEKvalekIQDCYSEESIRNILLEPKIMEAMVASPECLSYYGLDNIRsildyiskllge M5 "  "
CFPFFEAYASVLSGSRVWLYQELQAFDATAEEKVALEKIQDCYSEESIRNILLEPKIMEAMVASPECLSYYGLDNIRSILDYISKLLGE M6 "  "
cfpffeayasvlsgsrVWLYQELQAFDATAEEKvalekIQDCYSEESIRNILLEPKimeamvaspeclsyygldnirsildyiskllge F1 "  "
cfpffeayasvlsgsrvwlyqelqafdataeekvalekIQDCYSEESIRNILLEPKIMEAMVASPECLSYYGLDNIRSILDYISKllge F5 "  "
cfpffeayasvlsgsrVWLYQELQAFDATAEEKvalekIQDCYSEESIRNILLEPKIMEAMVASPECLSYYGLDNIRSILDYISKllge F6 "  "

mkgtlllallvtgelsfqttes
bg10
lvpffnvyasvlsgkrlyqelqtfnataevkvaleknlwll  M transcript
LVPFFNVYASVLSGKRlyqelqtfnataevkvaleknlwllenlrnillepqikeamiasqesqsnyevdnirSILDYISRllge M6 gel piece
r(8/13)                  i(30/33)       g(30/33)

  t(11/18/19)
mkgillllgllitgelsfqttea                              signal peptide part of the M transcript
  *
        g(19) v(19)                                            n(19)
        *     *
bg12
Cvpffevyasvlsgsrvwlyhelqsfdataeekvalekiqgcyreerlrnillepkimeamvaspecrsyysldnfrsildfi  M transcript
                                                                                     [continued]
cvpffegyasvvsgsrvwlyhelqsfdataeekvalekIQGCYREERlrNILLEPKIMEAMVASPECRSYYSLdNFRsildfisnllge M1 gel piece
cvpffegyasvvsgsrvwlyhelqsfdataeekvalekiqgcyreerlrNILLEPKIMEAMVASPECRSYYSLdNFRsildfisnllge M3 "  "
cvpffevyasvlsgsrvwlyhelqsfdataeekvalekiqgcyreerlrnillepkIMEAMVASPECRsyysldnfrsildfisnllge M4 "  "
cvpffevyASVLSGSRvwlyHELQSFDATAEEKvalekIQGCYREERlrnillepkIMEAMVASPECRsyysldnfrsildfisnllge M5 "  "
CVPFFEVYASVLSGSRVWLYHELQSFDATAEEKvalekIQGCYReerlrNILLEPKIMEAMVASPECRSYYSLDNFRsildfisnllge M6 "  "

lvtgelsfqttea (signal)
bg20
Clpffegyasvlsgsrvwlyqelqafnataeekvalekiqdcyseerirnillepkimeamvaspeclsyygldn  F transcript
     asvlsgsrvwlyqelqafnataeekvalekiqdcyseerirnillepkimeamvaspeclsyy  M transcript
CLPFFEGYASVLSGSRvwlyqelqafnataeekvalekIQDCYSEERirnillepkIMEAMVASPECLSYYGLDNIRSILDYISKllge M4 gel piece
CLPFFEGYASVLSGSRVWLYQELQAFNATAEEKVALEKIQDCYSEERIRNILLEPKIMEAMVASPECLSYYGLDNIRsildyiskllge M5 "  "
CLPFFEGYASVLSGSRVWLYQELQAFNATAEEKVALEKIQDCYSEERIRNILLEPKIMEAMVASPECLSYYGLDNIRSILDYISKLLGE M6 "  "
CLPFFEGYASVLSGSRvwlyqelqafnataeekvalekiqdcyseerIRNILLEPKIMEAMVASPECLSYYGLDNIRSILDYISKllge F6 "  "

lallmigelgfhttea (signal peptide)
bg24
cvpffagyag  M transcript
cvpffagyagvisgsrlwlyhelsafngtpketvayekiqdcykeqgvksqtlepqilasilvtpecl  M transcript
                   afngtpketvayekiqdcykeqgvksqtlepqilasilvtpeclqyyseetfft  F transcript
CVPFFAGYAGVISGSRLWLYHELSAFNGTPKETVAYEKIQDCYKEQGVKsqtlepqilasilvtpeclqyyseetfftkikdalkkisqh M1
cvpffagyagvisgsrLWLYHELSAFNGTPKETVAYEKIQDCYKEQGVKsqtlepqilasilvtpeclqyyseetfftkikdalkkisqh M2
cvpffagyagvisgsrLWLYHELSAFNGTPKetvayekIQDCYKEQGVKsqtlepqilasilvtpeclqyyseetfftkikdalkkisqh M3
CVPFFAGYAGVISGSRLWLYHELSAFNGTPKetvayekIQDCYKeqgvksqtlepqilasilvtpeclqyyseetfftkikdalkkisqh M4
CLPFFEGYASVLSGSRVWLYQELQAFNATAEEKVALEKIQDCYSEERIRNILLEPKIMEAMVASPECLSYYGLDNIRsildyiskllge M5
CVPFFAGYAGVISGSRLWLYHELSAFNGTPKetvayekIQDCYKEQGVKsqtlepqilasilvtpeclqyyseetfftkikdalkkisqh M6
CVPFFAGYAGVISGSRLWLYHELSAFNGTPKetvayekIQDCYKEQGVKsqtlepqilasilvtpeclqyyseetfftkikdalkkisqh F1
CVPFFAGYAGVISGSRLWLYHELSAFNGTPKETVAYEKIQDCYKEQGVKsqtlepqilasilvtpeclqyyseetfftkikdalkkisqh F2
CVPFFAGYAGVISGSRLWLYHELSAFNGTPKetvayekIQDCYKEQGVKsqtlepqilasilvtpeclqyyseetfftkikdalkkisqh F3

bg26 gailtlrrtflhgdlsqfyatvaervafekiqdcfreegqktiilnpq  M & F transcript
bg27 kilggnrlalnaylsmfqataaervafekiqdcfneepltktlkspqimmsilfsseckayypedsvnkma  M & F transcript
```

File S8. ***Abp*** **putative pseudogenes**

	Noncanonical splice site	Coding region coding disruption	both
Clade 1			a1p
Clade 2	bg3*, a5p, a28p	bg4p, bg5p,	a4p, bg6p, bg8p,
		bg28p, a6p, a8p,	a9p, bg30p, bg13p,
		bg9p, bg29p, a13p,	bg10*, a14p,
		bg14p, bg31p,	**bg15***, a16p,
		bg16p. bg32p,	**bg17***, bg33p, a22p,
		a21p, bg22p,	a23p
		bg23p, bg34p	
Clade 3			
Clade 4		bg25p	a25p
Clade 5	a26p	a30p	

***bg3** and **bg10** are expressed inspite of noncanonical splice sites and/or coding region disrutions

File S9. Data from qPCR analyses

Gland	Paralog	Avg Male CT value	ng total RNA	Male Protein Identification?	Paralog	Avg Female CT value	ng total RNA	Female Protein Identification ?	Male to Female Ratio ng total RNA	Sex specific (as per previous transcript data)
Lacrimal	Abpa2	15.81	0.21	Y	Abpa2	16.48	0.14	Y	1.5	N
Submax	Abpa2	*	n/a	N	Abpa2	37	7.94E-07	N	n/a	
Lacrimal	Abpbg2	18.08	0.035	Y	Abpbg2	18.9	0.022	Y	1.59	N
Lacrimal	Abpa3	16.43	0.026	Y	Abpa3	24.6	2.05E-05	N	1.27E+03	Y
Lacrimal	Abpbg3	22.5	0.096	Y	Abpbg3	*	n/a	N	n/a	Y
Lacrimal	Abpbg7	16.24	0.05	Y	Abpbg7	22.46	1.44E-03	Y	37.90	N
Lacrimal	Abpbg10	17.82	0.01	Y	Abpbg10	24.15	1.22E-04	N	85.55	Y
Lacrimal	Abpbg12	20.76	0.13	Y	Abpbg12	37.15	2.05E-05	N	6.48E+03	Y
Lacrimal	Abpbg20[b]	25.42	0.09	Y	Abpbg20[b]	29.42	3.49E-03	Y	26.26	N
Lacrimal	Abpa24[a]	19.26	0.02	N	Abpa24[a]	19.02	0.02	N	0.86	N
Lacrimal	Abpbg24[b]	14.61	0.02	Y	Abpbg24[b]	14.59	0.02	Y	0.93	N
Submax	Abpbg24	31.87	5.01E-09	N	Abpbg24	30.06	7.24E-09	N	0.69	
Lacrimal	Abpbg26[b]	*		N	Abpbg26	*		N		N
Submax	Abpbg26[b]	16.84	0.04	Y	Abpbg26[b]	16.22	0.06	Y	0.67	N
Lacrimal	Abpa27	38.05	7.76E-07	N	Abpa27	37.08	1.49E-06	N	0.52	N
Submax	Abpa27	20.59	0.09	Y	Abpa27	21.16	0.06	Y	1.47	N
Lacrimal	Abpbg27	32.39	7.26E-07	N	Abpbg27	30.85	2.27E-06	N	0.32	N
Submax	Abpbg27	18.48	0.02	Y	Abpbg27	19.03	1.43E-02	Y	1.50	N
Lacrimal	GAPDH[b]	24.13	0.27		GAPDH[b]	21.85	1.21		0.22	N
Submax	GAPDH[b]	22.22	1.05		GAPDH[b]	25.49	0.11		9.81	N

*undetectable Ct value

[a] Run at 60º

[b] Run at 63º

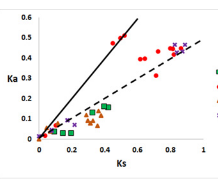

0.6
0.5
0.4
0.3
0.2
0.1
0
Ka
0
0.2
0.4
0.6
0.8
1
Ks
Abpa
Abpbg
Non-expr Abpa
Non-expr Abpbg

File S10.
